# Chimpanzees (*Pan troglodytes*) Produce the Same Types of ‘Laugh Faces’ when They Emit Laughter and when They Are Silent

**DOI:** 10.1371/journal.pone.0127337

**Published:** 2015-06-10

**Authors:** Marina Davila-Ross, Goncalo Jesus, Jade Osborne, Kim A. Bard

**Affiliations:** 1 Centre for Comparative and Evolutionary Psychology, Psychology Department, University of Portsmouth, Portsmouth, United Kingdom; 2 Department of Anthropology, University College London, London, United Kingdom; University of Florence, ITALY

## Abstract

The ability to flexibly produce facial expressions and vocalizations has a strong impact on the way humans communicate, as it promotes more explicit and versatile forms of communication. Whereas facial expressions and vocalizations are unarguably closely linked in primates, the extent to which these expressions can be produced independently in nonhuman primates is unknown. The present work, thus, examined if chimpanzees produce the same types of facial expressions with and without accompanying vocalizations, as do humans. Forty-six chimpanzees (*Pan troglodytes*) were video-recorded during spontaneous play with conspecifics at the Chimfunshi Wildlife Orphanage. ChimpFACS was applied, a standardized coding system to measure chimpanzee facial movements, based on FACS developed for humans. Data showed that the chimpanzees produced the same 14 configurations of open-mouth faces when laugh sounds were present and when they were absent. Chimpanzees, thus, produce these facial expressions flexibly without being morphologically constrained by the accompanying vocalizations. Furthermore, the data indicated that the facial expression plus vocalization and the facial expression alone were used differently in social play, i.e., when in physical contact with the playmates and when matching the playmates’ open-mouth faces. These findings provide empirical evidence that chimpanzees produce distinctive facial expressions independently from a vocalization, and that their multimodal use affects communicative meaning, important traits for a more explicit and versatile way of communication. As it is still uncertain how human laugh faces evolved, the ChimpFACS data were also used to empirically examine the evolutionary relation between open-mouth faces with laugh sounds of chimpanzees and laugh faces of humans. The ChimpFACS results revealed that laugh faces of humans must have gradually emerged from laughing open-mouth faces of ancestral apes. This work examines the main evolutionary changes of laugh faces since the last common ancestor of chimpanzees and humans.

## Introduction

The ability to use facial expressions and vocalizations flexibly has a significant impact on how humans communicate and it must have presented a foundation for a more comprehensive communication system throughout evolution [1–3]. This ability to flexibly combine facial and vocal expressions leads to a more explicit form of communication via versatile means [4] and contributes to the communicative tool kit of humans. For instance, people may show laugh faces while speaking or laughing, and they may also produce them silently, corresponding to a facial expression that may provide the individual with advantages, e.g., in emotional intelligence (see [5]).

However, little is known about the extent to which nonhuman primate facial expressions, which are also often closely linked to vocalizations [6–8], can be produced independently from vocalizations, despite the growing research interest in nonhuman primate multimodal communication (see [9–11]). Therefore, the present work tested whether nonhuman primates are able to produce their facial expressions in such a flexible way, by assessing whether their facial expressions are morphologically constrained by accompanying vocalizations. This study focused on chimpanzees (*Pan troglodytes*) and their open-mouth faces, which may, or may not, be accompanied by laugh sounds during spontaneous social play. Since chimpanzee laugh sounds have been phylogenetically linked to human laugh sounds [12, 13], another important aim of this study was to systematically examine the evolutionary relation (i.e., shared ancestry) of chimpanzee open-mouth faces and human laugh faces.

For the facial expression flexibility part of this study, we tested if chimpanzees produce the same types of open-mouth faces with laughter and without laughter. It is feasible that open-mouth faces may, or may not, be accompanied by laughter, i.e., either is motorically possible, and facial expressions may be produced under some motor control (albeit not necessarily intentional control) [7,14,15]. For the detailed description of open-mouth faces, ChimpFACS was used, which is a standardized facial coding system developed to measure facial movements of chimpanzees [16], based on FACS for humans [17,18]. With this non-invasive coding method, single facial movements based on known underlying musculature can be measured [16–19]. Both the facial movements and the underlying musculature can be shared by chimpanzees and humans, allowing a direct comparison of the facial muscle movements of chimpanzee and human facial expressions [19].

Furthermore, how chimpanzees use open-mouth faces with and without laugh sounds during social play was examined, since behaviours of different modalities are likely to have different roles in primate social communication. For example, vocalizations may help get the attention of others when the targetted recipients are looking away [9] or when they are too far away to see a visual stimulus [20]. In the present work, the occurences of open-mouth faces with and without laughter were compared when physical contact was present or absent during social play. Previous research suggests that open-mouth faces and laugh sounds differ in function as chimpanzees emit the former during both social and solitary play [21–23], but the latter predominantly during social play [24,25]. Additionally, the matching of open-mouth faces with and without laughter among the playmates was compared. Studies previously showed that nonhuman primates match the expressions of their playmates [24,26–28].

For the evolutionary reconstruction part of this study, the ChimpFACS data were used to specifically examine if the chimpanzees show the same facial movements when producing laugh sounds that characterize human laugh faces, based on previous FACS findings [29–32]. We find it reasonable to hypothesize that human laugh faces directly evolved from open-mouth faces of ancestral apes via gradual morphological and functional changes since great apes open their mouths widely while producing laugh sounds [33,34] and since great ape laugh sounds share ancestry with human laugh sounds based on phylogenetic analyses [12,13]. Alternative predictions are less parsimonious as they convey additional evolutionary changes (see [35]). The testing of the hypothesis is particularly relevant as van Hooff [21] claimed that the silent bared-teeth display replaced the open-mouth face of ancestral apes by converging with laughter, prior to the emergence of laugh faces of humans. His pioneering work has had notable impact on this research topic for more than four decades (see [36,37]), but it was not based on the detailed facial movements and knowledge of facial musculature with which we can now evaluate the morphologies of chimpanzee and human facial expressions, and compare them with and without laughter.

## Material and Methods

### Subjects

The subjects were 46 chimpanzees (24 females) of the Chimfunshi Wildlife Orphanage, Zambia. They lived in four semi-wild colonies, with 11–50 members per colony, situated within a naturally developed miombo woodland forest. Their ages ranged from 2 to 35 years (8 infants, 24 juveniles, 14 adolescents/adults). About half of the chimpanzees were wild-born and brought as orphans to this sanctuary, the rest of the chimpanzees were born in the sanctuary. The chimpanzees live in fission-fusion systems within settings that are ecologically similar to those of wild-born chimpanzees (see [38]).

The chimpanzee colonies were allocated four large outdoor enclosures, with the largest colony (50 chimpanzees) living in the largest enclosure (77 hectares), and the smallest colony (11 chimpanzees) living in the smallest enclosure, which was still very large (2 hectares). All colonies included both males and females, as well as infants, juveniles and adults. During the main feeding, around noon each day, most of the chimpanzees stayed indoors for about two hours, with families and/or subgroups in separate spaces to minimize the species-typical aggression that can surround feeding. During the other 22 hours each day, the chimpanzees remained in their larger social groups, in the very large outdoor enclosures. During the data collection period, the chimpanzees ate mostly fruits and vegetables that were bought from the local farmers, for example, strychnos fruits, sweet potatoes, sugar cane and cabbage. The food was more or less evenly distributed across the colonies and across the families/subgroups. Environmental enrichment was fully provided by the natural physical and social environments; additional enrichment was not necessary as they lived in semi-wild conditions.

The data reported in our manuscript were collected from June to August 2007. The manager of Chimfunshi at that time, Ms. Sylvia Jones, gave full permission to MDR to collect data. Chimfunshi is a sanctuary accredited by the Pan African Sanctuary Alliance (PASA), which means that it is inspected regularly and housing, veterinary care, husbandry, enrichment, and research adheres to the PASA guidelines and regulations. The data collection was carried out in agreement with the University of Portsmouth Psychology Research Ethics Committee.

### Data collection and video analysis

Spontaneous play between two playmates was video-recorded by an observer standing within 10 meters, a distance where chimpanzee laughter can be heard. A total of 1270 open-mouth faces were identified on the videos, based on the wide parting of the lips. They were coded as a single open-mouth face if they had no closed-mouth gaps of 0.5 seconds or longer. Forty-four subjects produced 697 open-mouth faces with laughter, 41 subjects produced 573 silent open-mouth faces.

For every open-mouth face, the play context of the subject was coded as either rough play or gentle play, and either with or without physical contact; rough play with contact could be hitting, rough play without contact could be chasing, gentle play with contact could be hand playing, and gentle play without contact could be a playful approach. Inter-coder reliability was good to excellent for open-mouth faces (Kappa = 0.75; 190 or 15% of open-mouth faces were assessed) and play contexts (Kappa = 0.86; 190, or 15%, of play contexts were assessed). Laugh bouts were previously identified and the coder reliability was excellent (Kappa = 0.84; see [12, 23]).

The video-coding of behaviour onsets and offsets was conducted with a precision of up to 0.12–0.16 seconds by using Interact 8 (Mangold, Arnstorf, Germany). The statistical analyses were computed with SPSS Statistics 20 (IBM, Chicago, IL, USA). Videos on 412 dyadic play bouts were obtained with a Sony HandyCam DCR-TRV19E (Sony Electronics, Oradell, NJ, USA). Play bouts started with the onset of the first play contexts and ended with the offsets of the last play contexts; they could include play gaps of 10 seconds or less. For the main analyses, the open-mouth faces with laughter and the silent open-mouth faces were compared in their facial morphology and in their contextual use.

### Morphology of open-mouth faces

ChimpFACS analysis [16] was applied to measure the muscle-based movements of open-mouth faces. For each open-mouth face, the presence of single visible facial movements (action unit, AU) was identified; some identified movements were based on undefined underlying muscles (action descriptors, AD). The following action units/descriptors were selected for the analysis based on previous chimpanzee research [23,34,39,40], the relevance of corresponding movements in human laughter research [29–32], as well as personal observations made by Davila-Ross and Bard during chimpanzee play: Raising of the cheeks (AU6), raising of the upper lips (AU10), pulling of the lip corners upwards and backwards (AU12), pressing down of lower lips (AU16), protrusion of the tongue (AD19), stretching of the lips (AU20, Risorius), opening of the lips (AU25), dropping of the jaw (AU26), and stretching of the jaw (AU27), and relaxing of the lower lip (AD160).

One hundred fifty silent open-mouth faces of 17 subjects revealed sufficiently high-quality visibility for the coding of action units and descriptors. They were matched with the same number of open-mouth faces with laughter, produced by 21 subjects. At the mid frames of these open-mouth faces, 854 action units and action descriptors were identified. A certified ChimpFACS coder (naïve about the aims of this study) first coded 140 open-mouth faces. The coder did not notice any facial movements that corresponded to action units/descriptors other than the ones decided on prior to the coding. The remaining open-mouth faces were then coded by a second ChimpFACS coder (with sounds turned off). An inter-rater reliability test was conducted and the agreement was excellent (Kappa = 0.87; 40 open-mouth faces were assessed).

### Contextual use of open-mouth faces

To examine the contextual use of open-mouth faces with laughter and silent open-mouth faces, two methods were applied. Initially, the occurrences of open-mouth faces with laughter and silent open-mouth faces were measured across social play contexts. The number of open-mouth faces was counted for every subject during rough play and gentle play, and when physical contact was present or was absent.

Then, the matching of open-mouth faces with laughter and silent open-mouth faces was examined. An open-mouth face of a subject was reported to match an open-mouth face of the playmate if it started either during the playmate’s facial expression or within 1 second after the offset of the playmate’s facial expression. Matching was counted only when there was no open-mouth face for at least 5 seconds prior to the onset of the playmate’s facial expression and when both playmates showed the same play context (e.g., both showed gentle play with physical contact). These decision rules reduced the chance that factors other than the playmate’s facial expression could trigger open-mouth faces in the subjects (e.g., a change from gentle play into rough play might trigger an open-mouth face). A total of 37 subjects were found to match the open-mouth faces of others. Hommel-Hochberg corrections were applied to adjust α levels for repeated statistical comparisons.

## Results

### Morphology of open-mouth faces

**Fig 1:**
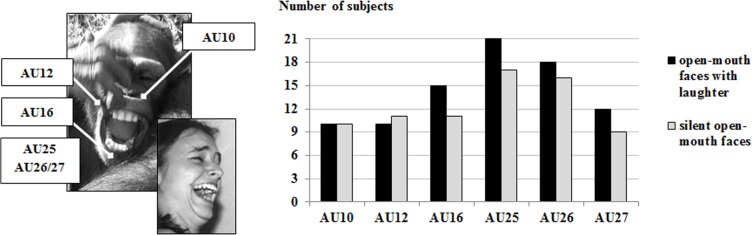
Action units of open-mouth faces. Total number of subjects producing the action units of open-mouth faces (with laughter and silently). AU10: Pulls the upper lip toward the nose, exposing the upper teeth; based on action of *levator labii superioris*; AU12: Pulls the lip corners upwards and backwards; based on action of *zygomatic major*; AU16: Depresses the lower lip, exposing the lower teeth; based on action of *depressor labii inferioris*; AU 25: Opens the lips; may involve action of different muscles (e.g., *depressor labii inferioris*, *levator labii superioris*, *orbicularis oris*); AU26 and AU27 are mutually exclusive. Prototypical open-mouth faces of chimpanzees consist of AU25 as well as AU26 and they may also include different combinations of AU10, AU12, and AU16 (for the action unit configurations, see Table 1). The individual in the picture has given informed consent.

Six action units were found to be common in ChimpFACS-coded open-mouth faces: Of 300 open-mouth faces, we found 19% contained AU10 (n = 56), 19% contained AU12 (n = 56), 43% contained AU16 (n = 128), 100% contained AU25 (n = 300), 83% contained AU26 (n = 249), and 17% contained AU27 (n = 50). Fig 1 shows the number of subjects that produced these action units with laughter and silently, respectively. Only 2 AUs were exhibited by more chimpanzees than expected by chance (given a chance value of. 5), AU25 (binomial value *p*<.001; by definition, an open mouth required the lips parted), and AU26 (binomial value *p*<.001). All of these AUs were produced by the same number of subjects with and without laughter (all chi-square values were non-significant).

It is important to note, however, that none of the AUs were produced in isolation. All AUs were found to be part of a configuration of facial movements consisting of between two and five AUs (Table 1). Table 1 shows the17 different action unit configurations that were found in the 300 open-mouth faces of this study. Fourteen of the configurations occurred both in open-mouth faces with laughter and in silent open-mouth faces. The occurrence patterns of these configurations were similar when comparing the open-mouth faces with laughter and the silent open-mouth faces: For both types of open-mouth faces, the parted lips with a jaw drop (AU25+26) and the parted lips with a jaw drop and a depressed lower lip (AU16+25+26) were the most frequent configurations (28% and 29% for open-mouth with laughter, respectively and 40% and 19% for silent open-mouth, respectively); the next four most frequent configurations accounted for a total of 36% of the open-mouth faces with laughter and 26% of the silent open mouth faces (7–12 occurrences for each configuration). Each of the remaining eight configurations had 6 or fewer occurrences. For further details on the data, see S1 Dataset.

**Table 1:** Action unit configurations.

**Types of open-mouth faces found in both modalities**	**Number of open-mouth faces with laughter (number of subjects)**	**Number of silent open-mouth faces (number of subjects)**
AU10+12+16+25+26	3 (2)	2 (2)
AU10+12+16+25+27	2 (2)	1 (1)
AU10+12+25+26	4 (2)	3 (2)
AU10+16+25+26	3 (2)	3 (2)
AU10+16+25+27	5 (4)	3 (1)
AU10+25+26 ^b^	11 (7)	12 (6)
AU10+25+27	2 (2)	1 (1)
AU12+16+25+26 ^b^	11 (7)	7 (5)
AU12+16+25+27	2 (1)	2 (2)
AU12+25+26 ^b^	7 (5)	9 (6)
AU16+25+26 ^a^	44 (13)	28 (9)
AU16+25+27	5 (3)	6 (4)
AU25+26 ^a^	42 (13)	60 (15)
AU25+27 ^b^	7 (6)	11 (6)
**Types of open-mouth faces found in one modality**	**Number of open-mouth faces with laughter (number of subjects)**	**Number of silent open-mouth faces (number of subjects)**
AU10+12+25+27	1 (1)	0
AU12+25+27	0	2 (2)
AU16+25	1 (1)	0

Types of open-mouth faces produced by the subjects for both modalities (produced both with laughter and silently) and for one modality (produced either with laughter or silently).

^a^ Refers to the two most common configurations.

^b^ Refers to the next four most common configurations

Four infrequent action units/descriptors were found in the ChimpFACS-coded open-mouth faces of the chimpanzees: AU6 (n = 1 open-mouth face), AD160 (n = 3), AD19 (n = 5), and AU20 (n = 6). They were excluded from further analyses and not presented in the configurations.

### Contextual use of open-mouth faces

**Fig 2:**
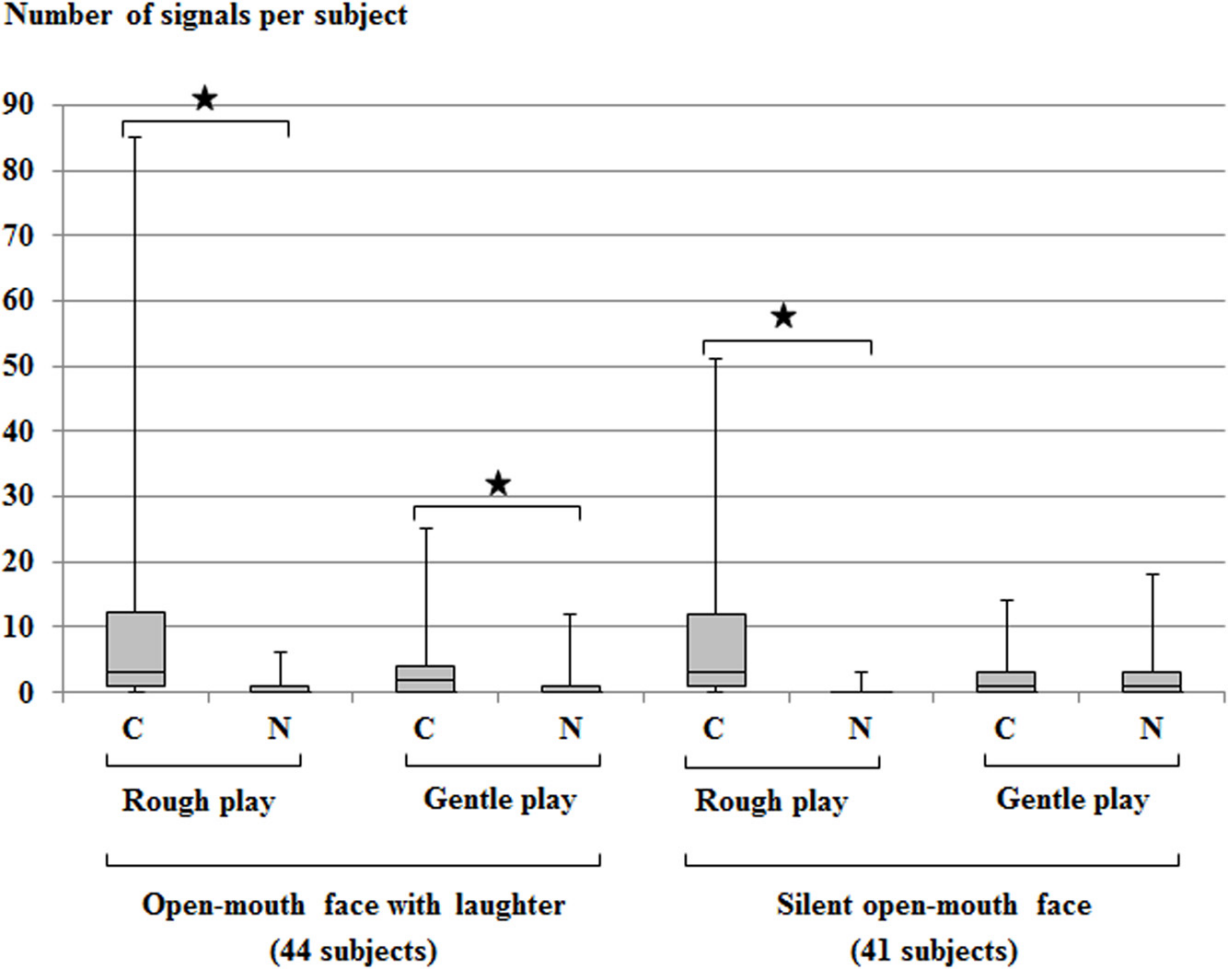
Open-mouth faces across play contexts. Number of open-mouth faces (with laughter and without laughter) produced by the subjects during rough and gentle play, with and without physical contact. The total number of subjects is shown in parentheses. C = play with physical contact; N = play with no physical contact. The box plots depict medians, upper and lower quartiles, and maximum and minimum range values.

Fig 2 illustrates the number of open-mouth faces produced by the chimpanzees during rough play and gentle play. During rough play, the chimpanzees produced significantly more open-mouth faces with laughter when in physical contact with the playmates than when there was no physical contact (two-tailed Wilcoxon Signed-Ranks with Hommel-Hochberg corrections, Z = -5.15, N = 44, p<.001). Such a difference was also found for gentle play (Z = -2.87, N = 44, p =. 003). During rough play, the chimpanzees also produced significantly more silent open-mouth faces when in physical contact with their playmates than when there was no physical contact (Z = -4.87, N = 41, p<.001). No such difference was found for gentle play (Z = -0.78, N = 41, p>.050). In other words, open-mouth faces with laughter were more often produced in play with physical contact than play without physical contact, for both rough and gentle play. Silent open-mouth faces were significantly more often found in play with physical contact than play without contact, but only during rough play; this association of silent open-mouth faces and physical contact was not found in gentle play. For further details on the data, see S1 Dataset.

**Fig 3:**
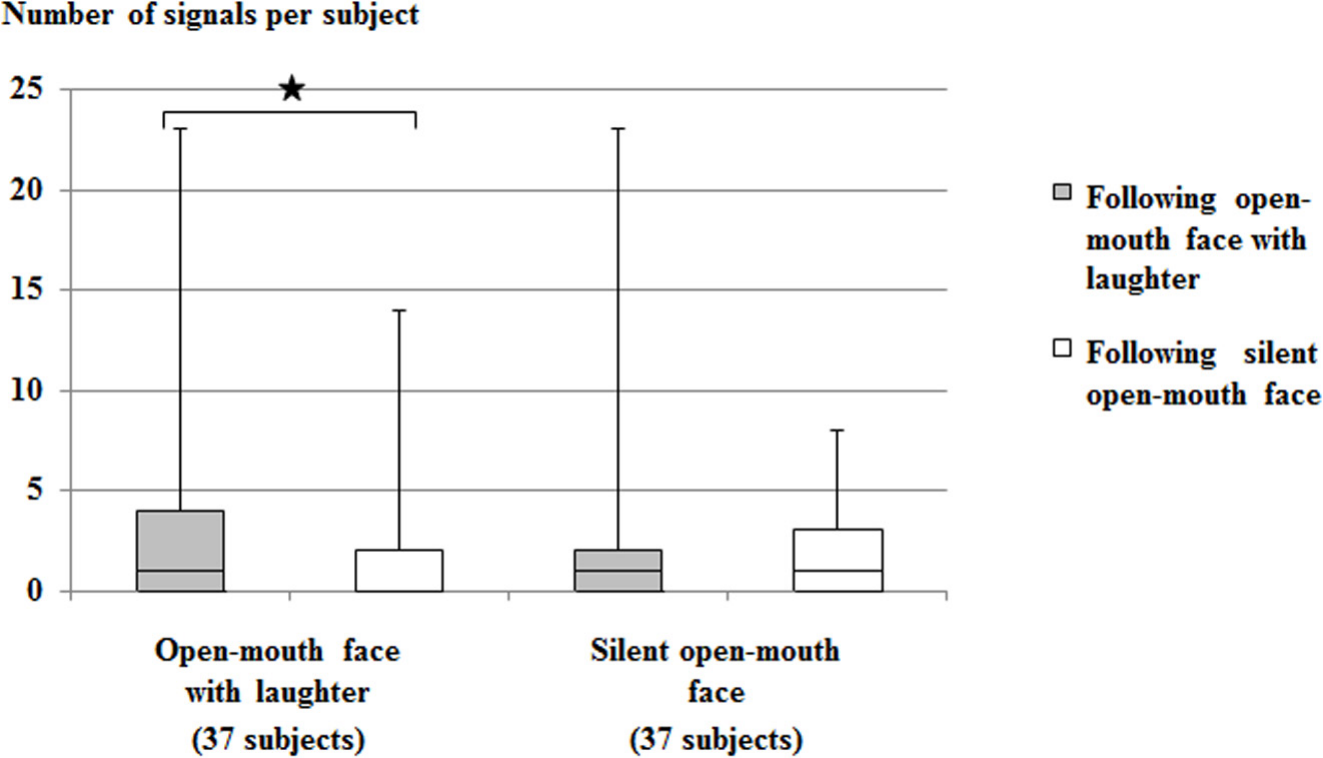
Matching of open-mouth faces. Number of matching open-mouth faces (with laughter and without laughter) of the subjects. The total number of subjects is shown in parentheses. The box plots depict medians, upper and lower quartiles, and maximum and minimum range values.

Fig 3 shows the matching of open-mouth faces by the chimpanzees. The playmates’ open-mouth faces with laughter were significantly more often followed by an open-mouth face with laughter of the subjects than the playmates’ silent open-mouth faces (two-tailed Wilcoxon Signed-Ranks with Hommel-Hochberg corrections, Z = -2.06, N = 37, p =. 039). No significant difference was found here for the silent open-mouth faces of the subjects (Z = -0.46, N = 37, p>.050). Therefore, open-mouth faces with laughter were significantly more often matched than not matched, unlike silent open-mouth faces.

## Discussion

The results on chimpanzee open-mouth faces and laughter revealed substantial commonalities with humans. The chimpanzees showed altogether 14 configurations of open-mouth faces that were produced both with laughter and without laughter. Furthermore, the frequency of occurrences for these facial configurations was similar when they were accompanied by laughter and when they were silent. These findings indicate that chimpanzees produce open-mouth faces with laughter without being morphologically constrained by the latter. Consequently, this study provides the first empirical evidence that a nonhuman primate species may produce facial expressions independently from closely associated vocalizations. In humans, the ability to be free of morphological constraints in facial movement leads to more explicit and versatile forms of communication [4].

Here we found that the chimpanzees used the facial and vocal behaviours differently while playing with their conspecifics. They produced the open-mouth faces together with laugh sounds mainly when in physical contact with their playmates and when matching their playmates’ open-mouth faces with laughter. These findings suggest that laughter accompanies open-mouth faces particularly in highly interactive social play. Added to the fact that chimpanzees produce laughter primarily when they play with a playmate instead of alone (see [24,25]), open-mouth faces with laughter arguably have more of a communicative function in chimpanzees than silent open-mouth faces. An alternative explanation is that play with playmates, particularly highly interactive social play, induces a higher state of arousal or joy in the chimpanzees, which may then lead to more laughter. Similarly, children laugh more in high arousal states [41]. We argue that the ability of chimpanzees to produce distinctive facial expressions independently from a vocalization, where the multimodal use affects meaning, may present the apes with the opportunity to communicate with their social partners in more explicit and versatile ways. Perhaps this ability represented a foundation for more complex forms of communication to have evolved closer towards the human lineage.

In addition, the evolutionary relationship of chimpanzee open-mouth faces and human laugh faces was examined. During laughter, the chimpanzees frequently showed three distinctive facial muscle activations that characterize human laugh faces [29,30], i. e., the pulling of the lip corners upwards and backwards (AU12), the parting of the lips (AU25), and the dropping of the jaw (AU26). Further muscle activations found in the laughing chimpanzee faces signified auxiliary movements in human laugh faces [29,31,32,42], including the raising of the upper lip (AU10), which may expose the upper teeth (see Fig 1). Such exposure of the teeth in laughing chimpanzees was, notably, not found by van Hooff [21]. The ChimpFACS data, thus, support the claim of fewest possible evolutionary changes, where laugh faces of humans must have gradually emerged in morph from open-mouth faces of ancestral apes (see [35,43–45]), not from silent-bared teeth displays of ancestral apes [21].

Future research should further investigate the link in production between facial expressions and vocalizations of nonhuman primates, as well as their phylogenetic relationship to humans for more insight on this research topic. We predict, based on the current findings, that the ability of humans to flexibly combine facial expressions with vocalizations evolved directly from such capability of ancestral apes. As social communication became more comprehensive [1–3], primordial laughter must have changed both in function and in form [12,13]. Ape-human comparisons in laughter suggest that the main changes involved exaggerations in the voicing [12,13] and in the raising of the cheeks (AU6) causing crow’s feet. The latter occurred rarely in the chimpanzees but is frequent in laugh faces of humans. This action unit represents a key attribute of the Duchenne smile [42], linked to 'felt' positive emotion [44], but this action unit is not necessary for a facial configuration to be indicative of a smile (see non-Duchenne smiles: [46]). Interestingly, research has shown that these phylogenetically more recent traits notably contribute to the potential impact that human laughter can have on others. Both the voiced laughter and the Duchenne laughter are perceived as more positive in comparison to the unvoiced laughter [47] and the non-Duchenne laughter [42, 48,49], respectively. Laughter must have had, therefore, an increasingly significant role in positive social interactions after the common ancestors of chimpanzees and humans, as this behaviour became more detached from the play context and emerged into a fundamental tool of language and emotional intelligence in humans [13,44,50].

## Supporting Information

S1 ARRIVE Checklist (PDF)

S1 Dataset Supplementary file of the Dataset. The number of occurrences of each action unit configuration for open-mouth faces with and without laughter for each chimpanzee subject, and the play contexts in which open-mouth faces occurred.
(PDF)
