# Supplementary material for: Chimpanzees (Pan troglodytes) Produce the Same Types of ‘Laugh Faces’ when They Emit Laughter and when They Are Silent
Source: PLoS One. 2015 Jun 10;10(6):e0127337. doi: 10.1371/journal.pone.0127337 (PMC4465483; doi:10.1371/journal.pone.0127337)
Supplement: S1 Dataset — The number of occurrences of each action unit configuration for open-mouth faces with and without laughter for each chimpanzee subject, and the play contexts in which open-mouth faces occurred. (PDF) [file pone.0127337.s002.pdf]

S1 Dataset: Supplementary file of the Dataset. The number of occurrences of each action unit configuration for open-mouth faces with and without laughter for each chimpanzee subject, and the play contexts in which open-mouth faces occurred.

Subject	OMF with laughter?	AU10+12+16+25+26	AU10+12+16+25+27	AU10+12+25+26	AU10+16+25+26	AU10+16+25+27	AU10+25+26	AU10+25+27	AU12+16+25+26	AU12+16+25+27	AU12+25+26	AU16+25+26	AU16+25+27	AU25+26	AU25+27	AU10+12+25+27	AU12+25+27	AU16+25
1	Yes						1					2	1					
1	No										2							
2	Yes													2	1			
2	No						2		2					2	2			
3	Yes													1				
3	No												1	4	1		1	
4	Yes					1					1	4		4				1
4	No													5				
5	Yes						1	1										
5	No						2			1		2		2				
6	Yes	2	1	2	2	1	1		2			5		7				
6	No	1										5	1	5				
7	Yes												3		1			
7	No														2			
8	Yes						3					1		1				
8	No						3					3		8				
9	Yes								2		1	6		5				
9	No		1	1		3	1		1			6	3	9				
10	Yes			2		1			1		2	1		7	1			
10	No							1			1	5		5	1			
11	Yes						1		2			7	1	1				
11	No													3				
12	Yes								1			6		2				
12	No													2				
13	Yes											1						
13	No				1							1		4	4			
14	Yes														1			
14	No										2			2				
15	Yes											2		6				
15	No	1							1		2	1		3				
16	Yes	1			1	2	3			2	2	3		2	1	1		
16	No			2			3		1	1	1	1	1	4	1		1	
17	Yes								1									
17	No																	
18	Yes										1			3				
18	No																	
19	Yes											1						
19	No																	
20	Yes		1				1		2			5		1				
20	No				2		1		2		1	4		2				
21	Yes							1							2			
21	No																	

		Laughter				Silent			
Age group	sex	rough contact	rough no contact	gentle contact	gentle no contact	rough contact	rough no contact	gentle contact	gentle no contact
**Adolescent**	**f**	0	2	8	5	1	1	1	5
**Juvenile**	**f**	2	0	2	1	4	0	4	5
**Juvenile**	**m**	31	5	12	12	22	0	13	18
**Juvenile**	**m**	3	0	2	0	0	0	1	0
**Adult**	**m**	12	0	3	1	23	0	4	3
**Adolescent**	**m**	9	1	5	7	12	1	2	4
**Juvenile**	**m**	13	0	1	3	51	0	1	1
**Juvenile**	**f**	4	0	4	0	14	0	3	10
**Juvenile**	**m**	23	4	3	12	4	0	1	0
**Juvenile**	**f**	19	0	11	1	3	0	3	3
**Adolescent**	**f**	20	0	0	1	2	0	0	0
**Juvenile**	**m**	3	1	3	0	3	0	0	2
**Juvenile**	**f**	3	2	3	4	17	3	2	7
**Juvenile**	**m**	2	0	5	1	1	0	0	0
**Juvenile**	**m**	49	6	0	4	15	1	4	0
**Juvenile**	**f**	46	1	25	9	20	1	14	17
**Juvenile**	**f**	17	0	12	1	7	0	0	2
**Juvenile**	**m**	17	0	0	1	18	0	7	4
**Juvenile**	**m**	5	1	4	0	2	1	5	2
**Juvenile**	**m**	85	3	12	5	44	2	13	10
**Infant**	**m**	3	0	6	2	1	0	2	5
**Infant**	**f**					3	0	3	2
**Infant**	**f**	3	0	1	0				
**Infant**	**f**	1	0	0	1	1	0	1	3
**Infant**	**f**	0	1	4	0	3	0	3	3
**Infant**	**m**	30	0	3	0	35	0	7	3
**Infant**	**m**	7	0	1	0	6	0	0	1
**Infant**	**m**	10	0	0	0				
**Juvenile**	**f**	2	0	0	0	8	0	1	0
**Juvenile**	**f**	1	0	0	0	1	0	0	1
**Juvenile**	**f**	1	0	2	0	1	1	0	0
**Juvenile**	**f**	1	0	1	1	4	0	0	0
**Juvenile**	**f**	0	0	3	0	0	0	1	0
**Juvenile**	**m**	5	0	1	1	0	0	1	0
**Juvenile**	**m**	0	0	1	0	0	0	1	0
**Juvenile**	**m**	0	0	1	0	0	0	2	2
**Adolescent**	**f**	4	0	2	1	2	0	3	1
**Adolescent**	**f**	0	0	0	1	0	0	0	1
**Adolescent**	**f**	4	1	1	0	6	0	0	1
**Adolescent**	**f**	1	0	0	0	1	1	0	1
**Adult**	**f**	1	0	0	1	0	0	0	0
**Adult**	**f**	5	0	0	1	2	0	0	0
**Adult**	**f**	0	0	5	0				
**Adult**	**m**	0	0	1	0				
**Adult**	**m**					4	0	0	0
**Adult**	**m**	2	0	0	0				
